# Knowledge, attitudes and practices of HIV-positive mothers regarding the benefits of exclusive breastfeeding at a regional hospital in the north east of Namibia

**DOI:** 10.4314/ahs.v21i3.15

**Published:** 2021-09

**Authors:** Daniel Opotamutale Ashipala, Getruida Shikukumwa, Medusalem Hangula Joel

**Affiliations:** Department of General Nursing Science, School of Nursing, Faculty of Health Science, University of Namibia (UNAM), Rundu, Namibia

**Keywords:** Knowledge, HIV, breastfeeding, attitudes, practices, HIV-positive mothers, Namibia

## Abstract

**Background:**

In sub-Saharan Africa, over 1,000 newborns are infected with HIV every day, despite available medical interventions. Mother-to-child transmission (MTCT) remains one of the primary sources of HIV infection in children and without interventions 40% of babies born from HIV-positive mothers would be infected with the virus. It is estimated that 300 000 children become infected with HIV worldwide, whilst 1.5 million children die when their mothers opt for other choices instead of breastfeeding.

**Objective:**

The purpose of the study was to assess and describe the knowledge, attitudes and practices of HIV-positive mothers regarding the benefits of exclusive breastfeeding at Rundu Intermediate Hospital, Kavango East Region in Namibia.

**Method:**

The study was a descriptive cross-sectional survey that used convenience sampling, as the researcher sought to use subjects available during the time of study to select 79 HIV positive mothers.

**Results:**

Participants in this study (94%; n=51) had good knowledge of the benefit of exclusive breastfeeding and that the benefits of breastfeeding outweigh the risk of HIV transmission from mother to child. The results confirmed that (77.2%; n=42) of the mothers opted to take ART with the babies until they stop breastfeeding.

**Conclusion:**

HIV positive mothers had good knowledge, attitudes and practices regarding the benefits of exclusive breastfeeding. A significant number of mothers were, however, not sure about breastfeeding exclusively for 6 months as they would stop if offered free formula milk for the baby. Support by the fathers and others in the community is vital.

## Introduction

The advent of the Human Immunodeficiency Virus (HIV) and the fact that it can be transmitted from mother to child through breastfeeding has brought a major public health concern to many low income countries with a high prevalence of HIV^1^. Mother-to-child transmission (MTCT) is a primary source of HIV infection in children and without interventions 40% of babies born to HIV-positive mothers would be infected with the virus^2^. In addition, in 2016 Africa recorded about 24% of pregnant women living with HIV to have limited access to ARVs to prevent MTCT services which resulted into 160,000 children becoming infected with HIV^3^, ^4^. Globally, about 300 000 children become infected with HIV each year of whom 1.5 million children die due to poor infant feeding options. This mostly results from economic and socio-cultural factors particularly in low income countries^5^. Based on that, the WHO^1^ further recommends exclusive breastfeeding for infants born to HIV-infected mothers. This includes several health benefits such as prevention of mother-to-child transmission of the HIV virus; and satisfying all the nutritional needs vital for the infants' physical growth and development. However, mothers living in high income settings can opt for exclusive replacement feeding, if formula feeding is acceptable, feasible, affordable, sustainable and safe^6^.

Sub-Sahara Africa has 90% of the 2.1 million global population of children aged below 15 years of age who are believed to be infected with HIV virus which is acquired mostly during pregnancy, delivery or postnatally^7^,^8^. Despite this, exclusive breastfeeding remains one of the most ideal and accepted traditional ways of feeding new-born infants which promotes infant healthy growth and development^7^. In addition, the rate of transmission in the region is believed to be higher, especially during the postnatal period where some HIV-positive mothers opt to cease breastfeeding exclusively due to pressure from traditional norms, lack of proper practical support and education on the breastfeeding practice^4^. However, the majority of African countries have developed health policies and guidelines which provide health advices to HIV- positive breastfeeding mothers on the 6-months exclusive breastfeeding period, introduction of supplementary feeds (post 6 months), as well as coverage of proper ART regimen to both mother and infant with an aim to lower chances of mother-to-child transmission^8^. Since the implementation of this PMTCT, approximately 14.8 million babies tested negative in 2018 of which 13.2 million were from sub-Saharan Africa^9^.

Ever since the world's first incidence of HIV was reported in 1986, Namibia has since adopted its first national PMTCT guideline in 2002 which was based on a single dosage of Nevirapine administered to HIV-positive mothers at the onset of labour, as well as a single dose given to the infant between 12 to 72 hours after delivery^1^. Thereafter, WHO established guidelines that provided the recommendation on the combined usage of regimens to enhance the efficacy of prevention. Based on that fact, Namibia saw the need to amend its first edition to incorporate the usage of other various treatment regimens for more effective PMTCT services^1^. In addition, Namibia amended and renamed its guidelines from PMTCT to Elimination of Mother-to-Child Transmission (EMTCT) which sought to eliminate the risk of virus transmission from mother to child during antenatal, labour and the postnatal period. Since then, the success of the PMTCT program in the country has brought about 60% reductions in new HIV infections amongst children^10^. These efforts were directed towards reaching the government's goal which is: “an end to HIV/AIDS epidemic by 2030” through administration of proper and effective HIV treatment regimens and exclusive breastfeeding of infants^11^. In addition, it is important that policy makers and program implementers understand the beliefs and customs surrounding infant breastfeeding to successfully implement PMTCT programs^1^. The Namibian eMTCT guidelines^11^ oblige health workers to promote and support breastfeeding as the best feeding option for every child born to an HIV-positive mother from Ante Natal Care (ANC). During the postnatal period a child can still be infected with acquired HIV from the mother through breast milk. None the less, studies have shown that exclusive breastfeeding, while the mother and child are on antiretroviral therapy (ART), further reduces the risk of transmission. According to the report on evaluation of Namibia's PEPFAR Operational Plan^12^, the country has made significant progress towards the elimination of MTCT and has acquired elimination status at the national level - the MTCT rate is 5%^11^.

Despite the efforts of the Ministry of Health and Social Services in the elimination of mother-to-child transmission, it seems Namibia's elimination of MTCT (eMTCT) is not winning the fight of eliminating the transmission from HIV-positive mothers to babies^12^. Babies who are born to HIV-positive mothers are at risk of acquiring HIV from their mothers through breastfeeding. However, child transmission of HIV reduces to less than 5% if the mother breastfeeds exclusively and keeps on antiretroviral treatment^13^. In 2009, the WHO^1^ released a new guideline, for the first time, recommending that HIV-positive mothers and their infants can both take antiretroviral drugs throughout the breastfeeding period until the child is 12 months old so as to reduce the risk of mother-to-child transmission of HIV up to less than 5%. Despite this step in the prevention of HIV transmission, the challenge of HIV from mother-to-child transmission remains mainly because of the strong culture of formula feeding within this community. According to Rundu Intermediate Hospital statistics of 2019 between April 2018 and March 2019, fifteen babies tested HIV positive during the 6^th^ week postnatal visit in Rundu District.

In view of the above, we carried out a study to assess and describe the knowledge, attitudes and practices of HIV-positive mothers regarding the benefits of exclusive breastfeeding at Rundu Intermediate Hospital, Kavango, East Region in Namibia.

## Materials and Methods

The study was a quantitative cross-sectional descriptive survey carried out at the Rundu Intermediate Hospital, Kavango East Region in the spring of September and October 2019. The population for this study was all HIV positive mothers who were admitted to the Rundu Intermediate Hospital, postnatal ward in Kavango East Region. According to the last population census survey of 2011 Rundu town has approximately 85,700 inhabitants. The minimum sample size was determined by using the formula for single proportion sample size, calculated as indicated by14 where n = is the sample size, N= is the total population and α= is the confidence limit 5% or 0.05. However, 79 HIV-positive mothers were sampled to allow for non-response and to increase the precision of the study. A convenience sampling technique was used to select participants that were available during the period of study. The study included all 79 HIV-positive mothers who were admitted at Rundu Intermediate Hospital postnatal ward at the time to ensure adequate representation. Ethical clearance was obtained from the Faculty of Health Sciences, specifically the School of Nursing Research and Ethics Committee (SoNREC 12/2019) and from the Ministry of Health and Social Services research review board reference numbers: 17/3/3 TGS before data collection. A pilot study was conducted, and the questionnaire was modified for any ambiguity. Thereafter, 79 questionnaires were distributed to participants by the resarcher to HIV-positive mothers who were admitted at Rundu Intermediate Hospital postnatal ward at the time of data collection. The questionnaire included the demographic profiles of participants and questions relating to knowledge of the respondents assessed the knowledge of HIV positive mothers regarding the benefits of exclusive breastfeeding. The attitudes of the respondents were assessed using Likert scale that ranged from strongly agrees to strongly disagree. In addition, their practices towards the benefits of exclusive breastfeeding were also assessed.

### Data analysis

Data was captured and analysed using the Statistical Package for Social Sciences (SPSS v.26). Descriptive statistics and frequency counts in percentages were used to present the findings. The questions were coded in the SPSS program; data were cleaned, compared and contrasted. The chi-squared test was used for categorical variables.

## Results

Of the 79 questionnaires administered, 54 were received. Out of this number, 25 questionnaires were rejected due to poor completion. The data analysis was done on 54 questionnaires. In this study gravidity was vital to be included as one of the demographic backgrounds since the study concerned mothers. The socio demographic characteristics of the respondents showed that 27 (50%) of the respondents who participated in the study were multigravida, followed by 15 (28%) who were grand multipara and 12(22%) primigravida. Almost all 51(95%) of the respondents were single. The age group distribution indicated that 25respondents (46.3%) were aged between 18 and 29 years old, while 11(20.4%) and 18(33.3%) accounted for those who were between the ages 30 to 34 and 35+ respectively.

The respondents confirmed that they received infant-feeding counseling during the antenatal period, and that the healthcare worker told them how to breastfeed correctly, and what exclusive breastfeeding entails. Most respondents also indicated that they were well informed that if both mother and infant take ARV during the breastfeeding period, the chances of HIV transmission from mother to child would be reduced. Another confirmation was that HIV positive and working mothers can still breastfeed exclusively by giving expressed breast milk. Although 38(63%) of the respondents indicated that a mother cannot introduce other food to an infant between the ages of 0 to 6 months, 17(31.5%) of women chose to differ, while only 3(5.6%) were unsure. This indicates that not all the women were informed about the introduction of other food to infants below 7 months of age. Most of the participants showed a vast knowledge on the benefit of exclusive breastfeeding.

**Table:** Chi-Square Tests

	P-Value	Df	Asymp. Sig. (2-sided)
Pearson Chi- Square	195.747^a^	18	.000
Likelihood Ratio	184.441	18	.000
No of Valid Cases	1002		

a 0 cells (0.0%) have expected a count less than 5. The minimum expected count is 8.60.

The probability of the chi-square test statistic (χ2 =195.747) was p<0.000, less than the alpha level of significance of 0.05. The research hypothesis that differences in knowledge are related to the rate is supported by this analysis. This indicates that there is statistically significant association between knowledge and the rate given by the mothers. Most of the mothers agreed to have knowledge concerning exclusive breastfeeding.

The HIV positive mothers believed that breastfeeding is the best option for their babies and that breastfeeding increases the bond between mother and child. Regarding the benefits of exclusive breastfeeding, most of the respondents indicated that stigma does not give them the fear of breastfeeding exclusively. About 59% (or 32) thought that benefits of breastfeeding outweigh the risk of HIV transmission from mother to child, while 10(18.5%) thought of the opposite and the rest were left unsure.

**Table:** Chi-Square Tests

	P-Value
Pearson Chi-Square	128.960^a^
Likelihood Ratio	127.241
No of Valid Cases	999

a 0 cells (0.0%) have expected a count less

b than 5. The minimum expected count is 12.49.

The Pearson Chi-Square statistic, χ2 = 128.960, and p<0.001; that is, an exceedingly small probability of the observed data under the null hypothesis of no relationship. The null hypothesis is rejected since p<0.05 (in fact p<0.001). The rate and attitude for the mothers seem to be related.

**Table:** Chi-Square Tests

	P-Value	df	Asymp. Sig. (2-sided)
Pearson Chi-Square	284.023^a^	12	.000
Likelihood Ratio	324.358	12	.000
No of Valid Cases	793		

a 0 cells (0.0%) have expected a count less than 5. The minimum expected count is 6.31.

The value of the test statistic is 284.023, degree of freedom=12. The corresponding p-value of the test statistic is p< 0.001. Since the p-value is less than the chosen significance level (α = 0.05), the null hypothesis is rejected and concludes that there is not enough evidence to suggest an association between the rate given to the practices on exclusive breastfeeding and the actual practices carried out by the mothers. Therefore, an association was found between the rate and the practices (χ2 (12)>= 284.023, p<0.001).

## Discussion

This study set out to assess and describe the knowledge, attitudes and practices of HIV-positive mothers regarding the benefits of exclusive breastfeeding at Rundu Intermediate Hospital, Kavango East Region in Namibia. The results found participants to be knowledgeable about the importance of breastfeeding exclusively despite being HIV positive. A mean percentage 77.2%(42) of participants agreed that if both mother and infant take ARV during the breastfeeding period it reduces the chances of HIV transmission and that the feeding counselling they acquired during the ANC period enables them to successively breastfeed exclusively for 6 months. The results speak to that of^14^ where participants demonstrated knowledge on prevention of mother-to-child HIV transmission through exclusive breastfeeding, as well as knowledge on breast milk as being sufficient for an infant's nutritional needs in the first six months of life. On the contrary,^4^ reported minimal knowledge of participants as to whether breast milk could prevent other infections such as diarrhoea.

Most participants (81.5%; n=44) demonstrated knowledge on milk expression specifically by HIV-positive working mothers. However, some mothers may opt against this practice, due to the doubts on its safety, acceptability and hygiene^1^. The respondents (79.3%; n=43) were knowledgeable about breast milk expression but only a few knew about the storage and use of expressed breast milk. Despite a total of (63%; n=34) participants voting against giving food to infants between 0 – 6 months, some participants indicated that it was ideal to do so. These results concurred with that of an Eastern Africa focussed systematic review as well as that of a studies done in Ghana and Brazil where participants demonstrated a vast knowledge on the importance of EBF and have known breast milk to be more important for the baby within the first six months and beyond^16^, ^17^, ^18^. Despite that, understanding the knowledge, attitude and practice towards EBF remains vital towards the identification of detriments which in return helps improve infant feeding practices among rural lactating mothers as a means of reducing infant morbidity and mortality^19^.

A positive attitude towards EBF was demonstrated by a (68.3%; n=37) mean percentile population of participants in this study. This is however higher in comparison with a somewhat positive attitude demonstrated in18, where an overall attitude median score added up to (39.9%; n= 26). Similarly, the results further concurred with that of^20^ who discovered (89%; n= 48) participants to have a positive attitude towards EBL. This study saw the importance of capturing the mothers' attitude towards the benefits of exclusive breastfeeding. Participants believed that exclusive breastfeeding is the best option for their babies and that breastfeeding increases the bond between mother and child. The results are however contrary to that of^21^, ^22^ where participants demonstrated a negative attitude towards exclusive breastfeeding with a fear that it might risk a transmission of the virus to their infants and further demanded a supply of formula milk from the government. In a study in Kenya, a positive attitude towards breastfeeding was associated with a good infant exclusive breastfeeding practice^23^. Most (70.4%; n=38) of respondents agreed that they had a fear of breastfeeding exclusively due to stigma. Similarly, the results tallied with that of the study in Ethiopia where (63.4%; n= 34) of participants desisted from EBF due to fear of stigma^24^. Furthermore, 70.4%(n=38) of participants in this study indicated that they had a fear of breastfeeding exclusively due to the belief that breastfeeding may cause a change in their body shapes, but agreed that breast milk alone can satisfy the baby for up to six months.

Most participants in this study (79.6%; n=43) concurred that breastfeeding establishes and strengthens the bond between the mother and the baby. The results tallied with the study done in China where EBF proved to have psychologically increased the bond between the mother and the baby through active communication, eye contact, and skin-to-skin touch^25^, ^4^.

The study discovered a favourable exclusive breastfeeding practice amongst study participants. This is evident of (96.3%; n=52) participants who indicated that they received Nevirapine prophylaxis for their babies after delivery and managed to give the first dose of Nevirapine to the baby within the first 24 hours of birth. However, about (24%; n=13) of the women under this study confirmed to have missed giving an ART dose in the past 7 days post-delivery. A similar detrimental practice was discovered in a study conducted in Burkina Faso where participants demonstrated a poor ART adherence and failure to exclusively breastfeed within the first six months of an infant's life^2^.

Satisfactorily, the study results showed that (59.3%; n=32) participants affirmed to breastfeed exclusively for 6 month. Consistent with a study done Lesotho, majority of the participants (89%) chose to breastfeed secondary to early infant feeding counseling and education given to them during antenatal period^26^. Despite that, EBF within the first six months of life remain crucial for all infants as it reduces infant and children mortality, as well as enhancing the general health of the baby^8^. Sadly, participants of a study done in Pakistan pointed to several factors hindering them from breastfeeding exclusively which include: insufficient milk syndrome, a mother's high workload, lack of social support, influences of culturally designated advisors, and the promotion and marketing strategies of infant formula companies^2^. It was further discovered in this study that the majority (76.9%; n=42) of participants opted not to cease breastfeeding exclusively within 6 months even if instructed by a spouse or family members. Furthermore, (77.8%; n=42) confirmed they were going to breastfeed on demand, whereas (11.1%; n= 6) mean percentile of participants opted otherwise. This practice is crucial for infants' cognitive development, reduction of starvation which in return minimizes the infant's levels of cortisol (a stress hormone), as well as physical maturity and development (milestones)^2^.

## Conclusion

The findings showed that the HIV positive mothers have good knowledge, attitudes and practices regarding the benefits of exclusive breastfeeding. The results have shown that the mothers were aware of the benefits of breastfeeding exclusively as well as the implications of opting for formula as an alternative. Mothers were also aware that HIV can be transmitted from mother to child and that these can be reduced if the advice provided by the health care workers is followed. With regards to attitudes, study participants were confident to share that they do not only have the knowledge of how to protect their babies from the virus, but they also had an attitude that ensures that the babies are protected as informed by the knowledge which they have gained. These are so held firmly that even family members or stigma do not influence their attitudes towards the protection of their babies. It is said practice makes perfect and this is what most of the HIV+ mothers did. They put into practice what they know about their situation and how to make the most of it. This was shared by the respondents who took the very first step of receiving Nevirapine prophylaxis for their babies and giving it to them within 24 hours of birth, and continuous feeling of breastfeeding their babies exclusively until 6 months old. A significant number of women were however not sure about breastfeeding exclusively for 6 months as they would stop if someone offers them formula milk for the baby.

HIV-education materials should have contents that can provide sufficient information to target audiences. The Ministry should also provide one-on-one mentoring on the benefits and the implications of breastfeeding for the HIV+ mother and the baby. Women in the study at hand should always be tested on the knowledge which was given on the previous follow-up as well, especially on what is practised and how they react to stigma or negativity from their families, partners and/or neighbours. The support of the partners is vital in this fight for the little one not to be infected. The role of the fathers of the children should therefore be involved, as well as the community at large.

## Figures and Tables

**Table 1:** Knowledge of HIV-positive mothers regarding the benefits of exclusive breastfeeding (n=54)

	Yes (%)	No (%)	Cannot remember (%)
Did you receive any infant-feeding counselling during the antenatal period?	88.9	5.6	5.6
Can HIV-infected mothers still breastfeed even if on ARVs?	70.4	22.2	7.4
If you are going to breastfeed, did the healthcare worker tell you how to breastfeed correctly?	72.2	11.1	16.7
Did the healthcare worker tell you what exclusive breastfeeding is?	63.0	16.7	20.4
Are you aware that there is a small chance that HIV can be passed from the mother to the baby during breastfeeding (on ART or not)?	61.1	27.8	11.1
Can a mother introduce other food to an infant between the ages of 0 to 6 months?	31.5	63.0	5.6
Do you know the benefits of exclusive breastfeeding?	77.8	13.0	9.3
Are you informed that if both mother and infant take ARV during the breastfeeding period the chances of HIV transmission from mother to child is reduced?	94.4	5.6	0.0
Do you know that giving formula to your infant puts them at risk of getting sick and the baby could die if it is not being prepared correctly?	64.8	27.8	7.4
Do you know that HIV positive and working mothers can still breastfeed exclusively by giving expressed breast milk?	81.5	14.8	3.7

**Table 2:** Attitudes of HIV-positive mothers regarding the benefits of exclusive breastfeeding (n=54)

	Yes (%)	No (%)	Not sure (%)
Do you think breastfeeding is the best option for your baby?	68.5	11.1	20.4
Do you think the benefits of breastfeeding outweigh the risk of HIV transmission from mother to child?	59.3	18.5	22.2
Are you going to make sure that you and the baby both continue to take ART until you stop breastfeeding?	77.8	9.3	13.0
Do you think breast milk only is enough for the baby up to six months?	70.4	13.0	16.7
Do you fear having to breastfeed exclusively because of stigma?	70.4	13.0	16.7
Does breastfeeding cause changes in the shape of the mother's body?	70.4	13.0	16.7
Should a mother be breastfeeding when a child has diarrhoea?	44.4	51.9	3.7
Is it true that breastfeed babies are less likely to suffer from malnutrition?	70.4	20.4	9.3
Bottle feeding is the fanciest way of infant feeding	72.2	27.8	0.0
Breastfeeding increases the bond between mother and child	79.6	13.0	7.4

**Table 3:** Practices of HIV-positive mothers regarding the benefits of exclusive breastfeeding (n=54)

	Yes (%)	No (%)	Not sure (%)
Have you received Nevirapine prophylaxis for the baby?	96.3	3.7	0.0
Did you give the first dose of Nevirapine to the baby within the first 24 hours after birth?	96.3	3.7	0.0
Have you missed any ART dose in the past 7 days?	24.1	74.1	1.9
Are you going to breastfeed your baby exclusively for 6 months?	59.3	24.1	16.7
Would you stop breastfeeding exclusively if someone offers to supply you with formula milk?	29.6	55.6	14.8
Would you stop breastfeeding if your partner, grandmother or neighbours ask you to?	11.1	79.6	9.3
How often are you going to feed the baby, every time the baby wakes up or cries (feed per demand)?	77.8	14.8	7.4
